# Association of immunophenotype with expression of topoisomerase II α and β in adult acute myeloid leukemia

**DOI:** 10.1038/s41598-020-62345-9

**Published:** 2020-03-26

**Authors:** Andrew P. Michelson, Shannon McDonough, Cheryl L. Willman, Eric R. Koegle, John E. Godwin, Stephen H. Petersdorf, Alan F. List, Megan Othus, Frederick R. Appelbaum, Jerald P. Radich, Mahrukh K. Ganapathi, Anjali S. Advani, Ram N. Ganapathi

**Affiliations:** 1 Cleveland Clinic, Cleveland, OH USA; 2 SWOG Statistical Center, Seattle, WA USA; 3 University of New Mexico Cancer Research and Treatment Center, Albuquerque, NM USA; 4 Providence Onc/Hem Care Clinic, Portland, OR USA; 5 Seattle Genetics Inc, Bothell, WA USA; 6 H. Lee Moffitt Cancer Center & Research Institute, Tampa, FL USA; 7 Fred Hutchinson Cancer Research Center, Seattle, WA USA; 8 Levine Cancer Institute, Carolinas Medical Center, Charlotte, NC USA

**Keywords:** Cancer, Cell biology

## Abstract

Anthracyclines used in the treatment of acute myelogenous leukemia (AML) inhibit the activity of the mammalian topoisomerase II (topo II) isoforms, topo II α and topo IIβ. In 230 patients with non-M3 AML who received frontline ara-C/daunorubicin we determined expression of topo IIα and topo IIβ by RT-PCR and its relationship to immunophenotype (IP) and outcomes. Treatment outcomes were analyzed by logistic or Cox regression. In 211 patients, available for analysis, topo IIα expression was significantly lower than topo IIβ (P < 0.0001). In contrast to topo IIα, topo IIβ was significantly associated with blast percentage in marrow or blood (P = 0.0001), CD7 (P = 0.01), CD14 (P < 0.0001) and CD54 (P < 0.0001). Event free survival was worse for CD56-negative compared to CD56-high (HR = 1.9, 95% CI [1.0–3.5], p = 0.04), and overall survival was worse for CD-15 low as compared to CD15-high (HR = 2.2, 95% CI [1.1–4.2], p = 0.02). Ingenuity pathway analysis indicated topo IIβ and immunophenotype markers in a network associated with cell-to-cell signaling, hematological system development/function and inflammatory response. Topo IIβ expression reflects disease biology of highly proliferative disease and distinct IP but does not appear to be an independent variable influencing outcome in adult AML patients treated with anthracycline-based therapy.

## Introduction

Standard induction therapy for acute myeloid leukemia (AML) consists of a combination of cytarabine (ara-C) and an anthracycline, such as daunorubicin or idarubicin^1,2^. While ara-C is a pyrimidine analogue that prematurely terminates DNA polymerization, the anthracyclines are inhibitors of topoisomerase II^3–6^. DNA topoisomerases relieve the topological constraints associated with the double-helical structure of DNA during vital DNA metabolic processes, including DNA replication, transcription, chromosome segregation and recombination^7^. Mammalian topoisomerase II (topo II) consists of two isoforms, topo IIα (M_r_ 170 kDa) and topo IIβ (M_r_ 180 kDa). Although the α and β topo II isoforms are highly homologous and catalyze similar biochemical reactions, they are genetically distinct and exhibit different patterns of expression and cellular distribution^7^. While expression of topo IIα is cell cycle-dependent^7^ topo IIβ levels remain unchanged during cell cycle progression^7–9^ and are maximal in terminally differentiated tissues^8,10,11^. This difference in expression suggests that these two isoforms exert distinct functional roles in cellular processes that require topological changes in the DNA molecule. It has been suggested that topo IIα may be important for DNA replication, whereas topo IIβ may be involved in cellular differentiation^8^. Using stable expression of shRNA targeted to topo IIα or topo IIβ, in cell culture models of human AML, we have been able to demonstrate a functional role for topo IIβ in apoptosis following all-*trans*-retinoic acid induced differentiation^12^. Further, our studies have demonstrated that clinically active drugs target different isoforms of topo II to exert their anti-tumor activity and that topo IIα and topo IIβ cooperate to maintain genome stability, which may be modulated by their C-terminal domain^13^.

Although topo II is a putative target of daunorubicin that is a key component of the induction treatment regimen for AML for over 3 decades, the significance of topo II α and β isoform expression and its association with other biomarkers related to outcome has not been satisfactorily addressed. Analysis of topo IIα expression have suggested correlations between gene amplification of topo IIα and response to anthracycline chemotherapy in breast cancer^14^. Some reports indicate no significant predictive value of topo IIα expression levels, while others suggest that topo IIα expression can predict treatment failure^15–19^. In contrast to the focus on topo IIα in AML the expression of topo IIβ that is targeted by daunorubicin and idarubicin has not been satisfactorily addressed. Topo IIβ expression has been proposed to have a role in resistance to drugs that target this isoform^20,21^ and in resistance to all-*trans* retinoic acid (ATRA) induced differentiation in M3 AML^22^. Interestingly, mitoxantrone that targets topo IIβ and other topo II inhibitors have been suggested to be involved in therapy related leukemia^23^. In the present study, we examined the expression of the topo II isoforms and possible relationships of topo IIα and topo IIβ expression to immunophenotype (IP) and outcomes in *de novo* and secondary adult AML blast samples from 230 patients enrolled in 4 SWOG studies who received ara-C/daunorubicin-based frontline chemotherapy.

## Results

### Correlation of topo IIα and topo IIβ expression with clinical characteristics and immunophenotype markers

**Figure 1:**
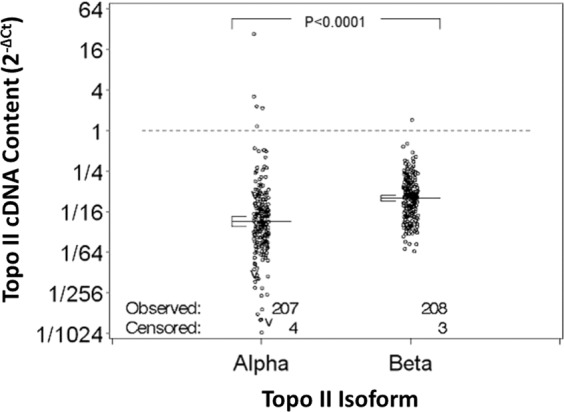
Topoisomerase II expression by topo II isoform in pre-treatment marrow specimens from 211 adult AML patients. The delta Ct (ΔCt) was calculated by subtracting the average cycle threshold (Ct) for each topo II isoform from the average β_2_-microglobulin (Ct). “V” indicates censored data. Solid lines show mean relative expression (ΔCt) and 95% confidence interval. Mean relative expression was 2.2-fold higher for topo IIβ compared to topo IIα.

Of the 230 treatment-naïve specimens available, topo II expression data from 211 patients was available for analysis. Patient and clinical characteristics for the 211 patients are summarized in Table 1. Expression (ΔCt) of the topo II isoforms was positively correlated (Fig. 1) and topo IIβ expression was on average 2.2-fold higher than topo IIα expression (CI 1.8–2.6, p < 0.001).

**Table 1:** Characteristics of 211 adult patients with previously untreated (N = 211) non-M3-AML.

Characteristics	Median	Range
Age (yrs)	64	19–88
Marrow blasts (%)	70	4–99
WBC (10^9^/L)	29.6	0.8–274
Peripheral blasts (%)	38	0–99
Hemoglobin (g/dL)	9.1	4.3–13.7
Platelets (10^9^/L)	58.5	2–1052
	Number	%
Sex:		
Female	94	45
Male	117	55
Race/Ethnicity:		
Native American	1	0.5
Asian, Pacific Islander	7	3
Black, African American	17	8
White/Hispanic	5	2
White/Non-Hispanic	179	85
Hispanic, NOS	2	1
AML Onset (months):		
De Novo	170	81
Secondary	41	19
FAB Class (local diagnosis):		
M1	47	22
M2	78	37
M4	46	22
M5	22	10
M6	1	0.5
M7	2	1
M0	9	4
Other	5	2
Performance Status		
0	63	30
1	104	49
2	25	12
3	17	8
Unknown	2	1
Cytogenetics Evaluated:		
Yes	169	80
No	42	20
Karyotype Category:*		
Normal	78	46
t(8:21)	7	4
inv(16)/t(16;16)	7	4
−5/del(5q)	7	4
−7/del(7q)	9	5
Other	9	5
Study		
S9031	75	36
S9126	10	5
S9333	81	38
S9500	45	21

*Percentages for karyotype categories are based on patients with cytogenetics evaluated.

Association of topo IIα or β expression with clinical characteristics is outlined in Table 2. Topo IIα expression was not found to be significantly associated with any patient or disease characteristic in univariate analyses. In contrast, topo IIβ expression was inversely associated with age (p = 0.001) and positively associated with both marrow and peripheral blast percentage (p < 0.001). Topo IIβ expression also varied significantly among FAB classes, being highest for M0 and lowest for M4 and M7 (p = 0.0012). Since immunophenotype is associated with outcome, analysis of the correlation of topo IIα or β expression with IP markers was carried out and is outlined in Table 3. Topo IIα expression was not correlated with any of the immunophenotypic markers measured. In contrast, topo IIβ expression was associated with expression of CD4 (p = 0.0025), CD7 (p = 0.01), CD11a (p < 0.0001), CD11b (p < 0.0001), CD11c (p = 0.007), CDw14 (p < 0.0001), CD15 (p = 0.045), CD16 (p = 0.009), CD34 (p = 0.03), CD54 (p < 0.0001) and HLA-DR (p < 0.0003). In multivariate analysis of 137 patients with complete data, topo IIβ expression was positively associated with age, as well as blood and marrow blast percentage (p < 0.001). Additional regression analysis of IP with outcome measures revealed event free survival was worse for CD56-ve compared to CD56-high (HR = 1.9, 95% CI [1.0–3.5], p = 0.04), and overall survival was worse for CD15-low as compared to CD15-high (HR = 2.2, 95% CI [1.1–4.2], p = 0.02). Neither topo IIα expression nor topo IIβ expression was significantly associated with clinical outcomes (CR, RD, RFS and OS) in uni- or multivariate analysis.

**Table 2:** Topo IIα and IIβ expression by clinical characteristics of 211 treatment-naïve adult non-M3 AML patients.

		Pts.	Topo IIα ΔCt			Topo IIβ ΔCt		
			^a^Mean	95% CI	P*	^a^Mean	95% CI	P*
Sex	F	94	4.6263	(4.2279, 5.0248)	0.26	3.3033	(3.0780, 3.5287)	0.62
	M	117	4.3317	(3.9978, 4.6656)		3.3768	(3.1880, 3.5656)	
Race	Asian	7	3.4503	(0.8010, 6.0996)	0.52	4.1253	(2.8630, 5.3877)	0.25
	Black	17	4.6634	(4.0769, 5.2499)		3.5518	(3.0479, 4.0557)	
	N.A./A.A.**	1	4.5682	NA		3.3044	NA	
	White	184	4.4968	(4.2205, 4.7730)		3.3020	(3.1487, 3.4553)	
	Unknown	2	3.1383	(−2.9872, 9.2638)		2.7353	(−2.7070, 8.1776)	
Hispanic	No	204	4.4853	(4.2234, 4.7472)	0.35	3.3421	(3.1947, 3.4895)	0.88
	Yes	7	3.8123	(2.7032, 4.9213)		3.4018	(2.5783, 4.2252)	
Secondary	No	170	4.4411	(4.1584, 4.7238)	0.73	3.2957	(3.1359, 3.4555)	0.18
AML	Yes	41	4.5534	(3.9369, 5.1700)		3.5445	(3.2064, 3.8826)	
SWOG	S9031	75	4.7364	(4.4125, 5.0603)	0.19	3.3004	(3.0788, 3.5220)	<0.0001
trial	S9126	10	5.1018	(3.5095, 6.6940)		4.1963	(3.5343, 4.8583)	
	S9333	81	4.3295	(3.8460, 4.8130)		3.5950	(3.3656, 3.8245)	
	S9500	45	4.1055	(3.5477, 4.6632)		2.7758	(2.4663, 3.0852)	
Year of	1992	32	4.8509	(4.4034, 5.2984)	0.24	3.4673	(3.1730, 3.7616)	0.076
entry	1993	35	4.6456	(4.0938, 5.1973)		3.1022	(2.7214, 3.4831)	
into	1994	11	5.2779	(4.0656, 6.4903)		3.9856	(3.2767, 4.6945)	
trial	1995	20	4.2586	(3.4299, 5.0874)		3.4189	(2.8697, 3.9680)	
	1996	51	3.9532	(3.3684, 4.5380)		3.2415	(2.9486, 3.5344)	
	1997	42	4.4320	(3.9085, 4.9554)		3.1622	(2.8130, 3.5114)	
	1998	20	4.6438	(3.3084, 5.9791)		3.7860	(3.3334, 4.2387)	
FAB	M0	9	4.5263	(2.6133, 6.4393)	0.85	2.4874	(1.6527, 3.3221)	0.0012
class	M1	48	4.3428	(3.9809, 4.7048)		3.0910	(2.8157, 3.3664)	
(local	M2	78	4.3830	(3.9286, 4.8374)		3.2699	(3.0202, 3.5196)	
diagnosis)	M4	46	4.6417	(3.9839, 5.2995)		3.7800	(3.4969, 4.0630)	
	M5	22	4.2870	(3.5581, 5.0158)		3.6301	(3.2513, 4.0088)	
	M6	1	5.6905	NA		3.0522	NA	
	M7	2	4.1100	(3.0501, 5.1699)		3.7085	(−0.892, 7.5062)	
	Other	5	5.2328	(2.6278, 7.8377)		3.5783	(1.8490, 5.3077)	
Cytogenetic	Favorable	14	4.2439	(2.6233, 5.8644)	0.66	3.7951	(3.1936, 4.3965)	0.34
risk group	Int-Normal	78	4.3503	(3.8822, 4.8184)		3.2937	(3.0178, 3.5696)	
	Int-II	51	4.7509	(4.3165, 5.1852)		3.4822	(3.2511, 3.7132)	
	Unfavorable	26	4.4781	(3.9370, 5.0193)		3.2704	(3.8841, 3.6566)	
		Pts	^b^Coeff.	95% CI	P*	^b^Coeff.	95% CI	P*
Age (years)		211	0.00633	(−0.0112, 0.0239)	0.48	0.0163	(0.0067, 0.0260)	0.001
Marrow blasts (%)		196	−0.00167	(−0.0141, 0.0108)	0.79	−0.0165	(−0.0230, −0.0101)	<0.0001
WBC (10^9^/L)		211	0.00089	(−0.0041, 0.0059)	0.73	−0.0009	(−0.0037, 0.0020)	0.55
Peripheral blasts (%)		202	0.00054	(−0.0079, 0.0090)	0.90	−0.0123	(−0.0167, −0.0078)	<0.0001
Hemoglobin (g/dL)		206	0.01830	(−0.1297, 0.1663)	0.81	−0.0048	(−0.0876, 0.0781)	0.91
Platelets (10^9^/L)		210	0.00157	(−0.0011, 0.0042)	0.25	0.0016	(0.0001, 0.0031)	0.04

Abbreviations: Pts. = patients; CI = confidence interval.

^a^Means (and 95% CI of the means), represent mean ΔCt calculated for the listed subgroup.

^b^Coeff. represents the coefficient (and associated 95% CI) from a univariate linear regression model with the ΔCt variable as the outcome and the listed variables as covariates. Coeff. greater than 0 indicates a positive association: as the covariate values increase, the ΔCt values increase on average. Coeff. less than 0 indicates a negative association; as the covariate values increase, the ΔCt values decrease on average.

*P-value for heterogeneity of mean ΔCt among categories or regression on continuous variables; calculated from univariate linear regression models with ΔCt as the outcome and each variable as a covariate.

**N.A./A.A.: Native America or Alaskan Native.

**Table 3:** Topo IIα and IIβ expression by immunophenotype markers of 186 treatment-naïve adult non-M3 AML patients.

Immunophenotype *		Pts.	Topo IIα ΔCt			Topo IIβ ΔCt		
			Mean	95% CI	P**	Mean	95% CI	P**
CD2	HIGH/LOW	10	4.2453	(3.3954, 5.0952)	0.67	2.8719	(1.9956, 3.7486)	0.14
	NEGATIVE	176	4.5147	(4.2180, 4.8114)		3.3844	(3.2255, 3.5432)	
CD4	HIGH	16	4.1362	(3.2256, 5.0468)	0.50	3.7466	(3.3509, 4.1423)	0.0025
	LOW	41	4.7695	(4.2694, 5.2695)		3.7716	(3.4116, 4.1316)	
	NEGATIVE	129	4.4598	(4.0953, 4.8242)		3.1766	(2.9935, 3.3597)	
CD7	HIGH	23	3.9183	(3.1032, 4.7334)	0.27	2.8087	(2.4433, 3.1742)	0.010
	LOW	21	4.8258	(4.1192, 5.5324)		3.6811	(3.2225, 4.1396)	
	NEGATIVE	140	4.5218	(4.1847, 4.8589)		3.4168	(3.2357, 3.5978)	
CD11A	HIGH	109	4.4966	(4.1603, 4.8328)	0.57	3.6105	(3.4184, 3.8027)	0.0002
	LOW	48	4.3216	(3.6616, 4.9816)		3.1290	(2.8229, 3.4351)	
	NEGATIVE	29	4.8095	(4.0099, 5.6091)		2.7802	(2.3649, 3.1954)	
CD11B	HIGH	41	4.8756	(4.3956, 5.3556)	0.30	4.0955	(3.8647, 4.3263)	<0.0001
	LOW	25	4.6374	(3.8819, 4.7239)		3.0996	(2.7450, 3.4541)	
	NEGATIVE	120	4.3434	(3.9628, 2.9839)		3.1580	(2.9567, 3.3593)	
CD11C	HIGH	94	4.5009	(4.1292, 4.8727)	0.88	3.5975	(3.3765, 3.8185)	0.007
	LOW	48	4.3987	(3.7782, 5.0193)		3.0564	(2.7458, 3.3669)	
	NEGATIVE	44	4.6093	(3.9745, 5.2442)		3.1704	(2.8697, 3.4710)	
CD13	HIGH	134	4.3263	(3.9917, 4.6609)	0.12	3.3823	(3.2031, 3.5615)	0.42
	LOW	34	5.0728	(4.4835, 5.6621)		3.4227	(3.0434, 3.8019)	
	NEGATIVE	18	4.7135	(3.5730, 5.8540)		3.0426	(2.4036, 3.6815)	
CDw14	HIGH	34	4.7128	(4.0369, 5.3887)	0.55	4.2883	(4.0132, 4.5633)	<0.0001
	LOW	17	4.8316	(3.9607, 5.7026)		3.7438	(3.2190, 4.2687)	
	NEGATIVE	135	4.4049	(4.0647, 4.7451)		3.0735	(2.9024, 3.2445)	
CD15	HIGH	97	4.4044	(4.0045, 4.8042)	0.79	3.5449	(3.3310, 3.7589)	0.045
	LOW	23	4.5911	(3.8205, 5.3617)		3.1579	(2.7509, 3.5649)	
	NEGATIVE	66	4.6094	(4.1177, 5.1010)		3.1497	(2.8760, 3.4233)	
CD16	HIGH/LOW	7	5.8795	(4.2576, 7.5014)	0.06	4.3967	(3.4498, 5.3454)	0.009
	NEGATIVE	179	4.4463	(4.1585, 4.7340)		3.3161	(3.1593, 3.4729)	
CD18	HIGH	121	4.3574	(4.0092, 4.7057)	0.06	3.4947	(3.3033, 3.6860)	0.058
	LOW	34	4.3074	(3.5520, 5.0629)		3.0955	(2.7007, 3.4902)	
	NEGATIVE	30	5.2622	(4.6282, 5.8963)		3.0977	(2.7122, 3.4831)	
CD19	HIGH/LOW	6	5.2026	(3.2847, 7.1204)	0.37	3.4779	(2.4885, 4.4673)	0.78
	NEGATIVE	180	4.4768	(4.1881, 4.7655)		3.3528	(3.1931, 3.5124)	
CD33	HIGH	159	4.4490	(4.1326, 4.7654)	0.59	3.3184	(3.1443, 3.4925)	0.50
	LOW	23	4.7134	(4.0052, 5.4216)		3.5973	(3.2237, 3.9708)	
	NEGATIVE	4	5.3101	(4.0324, 6.5878)		3.5012	(2.1531, 4.8492)	
CD34	HIGH	106	4.6027	(4.2370, 4.9685)	0.07	3.5299	(3.3438, 3.7159)	0.03
	LOW	6	2.6970	(0.6967, 4.6972)		2.7758	(1.5067, 4.0448)	
	NEGATIVE	74	4.4996	(4.0361, 4.9630)		3.1560	(2.8823, 3.4298)	
CD44	HIGH	182	4.4889	(4.2018, 4.7760)	0.74	3.3482	(3.1893, 3.5072)	0.40
	LOW/NEGATIVE	3	4.8630	(−0.9289, 10.655)		3.8840	(2.2043, 5.5638)	
CD54	HIGH-SUB	11	4.8624	(4.2678, 5.4571)	0.32	3.8084	(3.4275, 4.1893)	<0.0001
	HIGH-TOT	47	4.8973	(4.3107, 5.4840)		3.5599	(3.2425, 3.8773)	
	LOW-TOT	64	4.2672	(3.7429, 4.7916)		3.6311	(3.3984, 3.8638)	
	NEGATIVE	63	4.3619	(3.8810, 4.8428)		2.8482	(2.5649, 3.1315)	
CD56	HIGH	27	4.6836	(3.8938, 5.4734)	0.60	3.6942	(3.2065, 4.1818)	0.20
	LOW	5	3.7128	(0.1725, 7.2530)		3.4801	(2.1129, 4.8474)	
	NEGATIVE	154	4.4936	(4.1865, 4.8007)		3.2937	(3.1272, 3.4601)	
HLA-DR	HIGH	154	4.4355	(4.1422, 4.7287)	0.58	3.4844	(3.3177, 3.6511)	0.0003
	LOW	5	5.0998	(2.3465, 7.8532)		3.5153	(2.3476, 4.6829)	
	NEGATIVE	27	4.7585	(3.7570, 5.7600)		2.5996	(2.2039, 2.9952)	

Abbreviations: Pts = patients; CI = confidence interval.

*Expression of immunophenotype markers was originally classified as high in the total blast population (HIGH-TOT) or a subpopulation (HIGH-SUB), low in the total blast population (LOW-TOT) or a subpopulation (LOW-SUB), or negative. These categories were combined as needed to ensure adequate category sizes for comparisons when possible.

**P-value for heterogeneity of mean ΔCt among immunophenotype categories calculated from univariate linear regression models with ΔCt as the outcome and each immunophenotype marker as the covariate; means and 95% CIs of the means of ΔCt were calculated for each subgroup listed.

Data from analysis of P-glycoprotein expression and function, representing possible determinants of resistance was available and correlation with topo II isoform expression was assessed. Topo IIβ expression was weakly correlated with P-glycoprotein expression detected with the MM4.17 antibody (p = 0.07) and P-glycoprotein function based on rhodamine efflux (p = 0.04). Both parameters were inversely related to topo IIβ expression but not correlated with topo IIα expression. However, in univariate analysis neither P-glycoprotein expression or function was associated with OS, RFS or EFS.

### Pathway analysis of immunophenotype markers with topo IIα and topo IIβ

**Figure 2:**
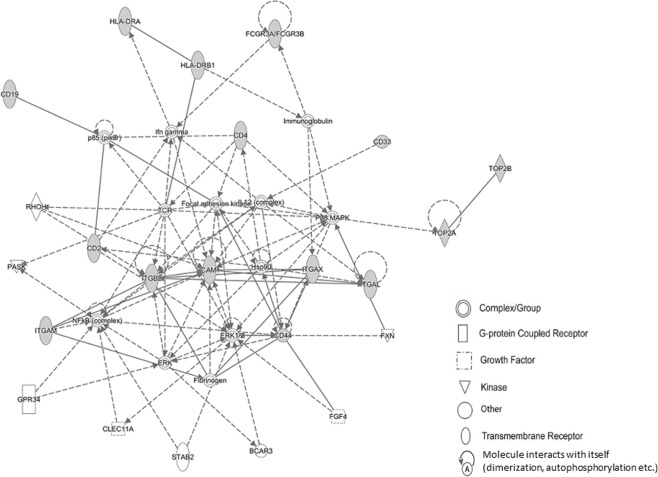
Ingenuity pathway analysis of drug pharmacodynamic targets, associated immunophenotype markers and topo II. The (—) and (---) lines in network represent direct and in indirect interaction, respectively. Gene ID is identified in the network symbols and symbol key describes biological relevance. Gray fill color in symbols identifies focus genes from the dataset.

**Figure 3:**
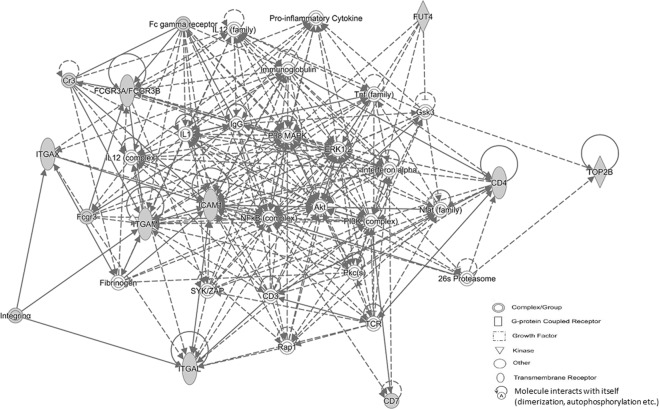
Ingenuity pathway analysis of topo IIβ and significantly associated immunophenotype markers. The (—) and (---) lines in network represent direct and in indirect interaction, respectively. Gene ID is identified in the network symbols and symbol key describes biological relevance. Gray fill color in symbols identifies focus genes from the dataset.

Ingenuity pathway analysis of drug pharmacodynamic targets, immunophenotype markers and topo II are outlined in Figs. 2 and 3. In Fig. 2, among 6 networks the major network with a score of 34 and 15 focus molecules identified the direct interaction of topo IIα/topo IIβ and cell-to-cell signaling, hematological system development/function and immune cell trafficking as the top diseases and functions. In Fig. 3, with a focus on topo IIβ and the significantly associated immunophenotype markers, among 3 networks the major network generated a score of 25 with 9 focus molecules and an association with cell-to-cell signaling/interaction, hematological system development/function and inflammatory response among the top diseases and functions. Overall, both networks identified similar functional events and disease states based on the interaction between significantly associated immunophenotype markers and topo II.

## Discussion

This is the first study examining the expression levels of both topo IIα and IIβ in a large cohort of de *novo* and secondary AML patients and evaluating associations between topo II isoform expression, clinical outcome, immunophenotype and other patient characteristics. Inhibitors of topo II, such as the anthracyclines, are the cornerstones of AML treatment and presumed primarily to target topo IIα. Despite the importance of the anthracyclines at inducing complete remission, it is unknown if the expression levels of topo IIα can predict clinical outcome. Present results do demonstrate a significant inter-individual variability in topo IIα mRNA levels and failed to show any significant association between topo IIα expression and any disease characteristic in *de novo* and secondary AML patients. It has been shown that exposure of AML blast cells to the anthracyline daunorubicin promotes expansion of topo IIα negative cells^19^. This *ex-vivo* observation, on daunorubicin-treatment dependent selection of topo IIα negative cells was however, not linked to clinical outcome. Despite active investigation into the clinical significance of topo IIα, little is known about the importance of topo IIβ in AML. Gieseler *et al*.^24^ reported that blast cells from patients with elevated activity of topo IIβ expressed reduced sensitivity *in vitro* to daunorubicin or idarubicin and relapse from treatment with anthracyclines may be linked to a significantly lower topo IIα/β ratio. Our studies in HL-60 cells with targeted stable down-regulation of topo IIα or β isoform or in models engineered to express either topo IIα or topo IIβ, indicate that while sensitivity to doxorubicin is unaltered, a 2- to 4-fold reduction in etoposide sensitivity is observed following down-regulation of the α isoform, and a marked decrease in sensitivity to amsacrine, idarubicin and mitoxantrone is seen in cells depleted of the β isoform^12,13^. However, topo IIβ was significantly associated with several factors that have been associated with favorable outcomes in AML, such as younger age, low CD4, CD14, CD16, CD54, CD11b, and HLA-DR, as well as with unfavorable factors, high peripheral and marrow blast percentage and increased CD7 expression. While reports on relationship or precise role of immunophenotype and prognosis in AML is controversial^25–29^, the association with topo IIβ but not topo IIα expression and proposed network of topo IIβ with immunophenotype markers suggests a potential role for topo IIβ expression and immunophenotype in the biology of AML. Song *et al*.^30^ reported high topo IIβ/topo IIα expression to be correlative with favorable outcome but this observation could not be compared with the present results since induction therapy utilized the anthracycline idarubicin and most patients also received hematopoietic stem cell transplantation. Recent reports^31,32^ on use of idarubicin in adult AML indicate: (a) that an increased cumulative dose of idarubicin during consolidation can improve leukemia-free survival; and (b) comparing idarubicin to high dose daunorubicin during induction did not indicate significant differences in CR rate, relapse and survival. Heterogeneity in development of resistant cells as well as differential expression in key pathways has been suggested to involved in refractory AML^33,34^. Analysis of topo II α and β expression coupled with immunophenotype in AML cells from patients with disease that is resistant to anthracycline/cytarabine therapy might provide insights on biomarkers relevant to outcome. In summary, topo IIβ expression reflects aspects of disease biology, such as highly proliferative disease (higher blasts) and immunophenotypic differences but does not appear to be an independent variable influencing outcome in adult AML patients treated with anthracycline-based therapy.

## Materials and Methods

### Patients and specimens

Bone marrow (BM) specimens were provided by the SWOG AML/MDS Repository for 230 adult patients with non-M3 AML by FAB criteria who were enrolled during 1992–1998 for ara-C/daunorubicin-based frontline chemotherapy on any of four SWOG studies S9031, S9126, S9333 and S9500. Specimens of cryopreserved BM cells and expected to contain >70% blasts, were used when available, otherwise RNA extracted from specimens upon receipt at the repository were used. All patients provided written informed consent in accordance with institutional and federal guidelines. The protocol was approved by the Cleveland Clinic IRB CC 937: S9031-S9126-S9333-S9500-B Topoisomerase 2 Expression and Acute Myeloid Leukemia (AML).

The study was performed in accordance with the Declaration of Helsinki.

### RNA extraction

RNA extraction was performed on the entire BM specimen using the Trizol reagent (Invitrogen USA) per the manufacturer’s instructions. The RNA pellets were re-suspended in 50 μL RNase-free water and stored at −80 °C.

### qRT-PCR

RNA (1500 ng) was reverse transcribed to cDNA and used for quantitative PCR reaction, which was carried out in triplicate at the Case Comprehensive Cancer Center, Gene Expression Core Facility using an ABI PRISM 7900HT (Martina Veigl, Director). The primers used for the PCR reaction were: topo IIα (forward: 5′-TGTCTCTCAAAAGCCTGATC-3′, reverse: 5′-GTCCATATGGAAGTCATCAC-3′), topo IIβ (forward: 5′-TAAAGGCCGAGGGGCAAAGA-3′, reverse: 5′-GCAGAGAAGGTGGCTCAGTA-3′) specific primers and β_2_-microglobulin (B2MG) (forward: 5′-CTTGTCTTTCAGCAAGGACTGG-3′ and reverse: 5′-CATGATGCTGCTTACATGTCTC-3′primers). B2MG was used as an endogenous control to normalize topo IIα and topo IIβ expression.

### Immunophenotypic and cytogenetic analysis

Immunophenotyping was performed at the SWOG AML/MDS Repository at the University of New Mexico (Cheryl Willman, Director). Blast cells were assessed for expression, of the following IP markers: CD2, CD4, CD7, CD8, CD11a, CD11b, CD11c, CD13, CDW14, CD15, CD16, CD18, CD19, CD33, CD34, CD38, CD44, CD54, CD56 and HLA-DR. Expression of these markers in the total population was characterized as negative, high, low, low/high or low/negative. Four other markers were assayed but excluded from this analysis: CD3, CD8 were negative for all patients; CD38 and CD45 were highly expressed in all but 2 patients. Cytogenetic studies of pretreatment marrow or peripheral blood were performed at SWOG-approved laboratories and centrally reviewed by the SWOG Cytogenetics Committee.

### Statistical and pathway analysis

The ΔCt for topo IIα or IIβ RNA was calculated by subtracting the average cycle threshold (Ct) for each topo II isoform from the average β_2_-microglobulin (Ct). Average topo II expression of 40 was considered right-censored. Topo IIα expression was censored on four observations and topo IIβ expression on three observations.

Clinical data (age, sex, race/ethnicity, secondary versus *de novo* AML onset, FAB classification, cytogenetics, marrow and peripheral blood blast percentages, WBC and PLT counts, and hemoglobin) and treatment outcomes were collected and evaluated per standard SWOG procedures as part of the clinical trials on which the patients participated. Complete response (CR) and resistant disease (RD) were defined by standard criteria^35^. Overall survival (OS) was measured from date of study entry until death from any cause, with observation censored at the date of last contact for patients last known to be alive. Relapse-free survival (RFS) was measured from the date of achieving CR until relapse or death from any cause, with observation censored at the date of last contact for patients last known to be alive without report of AML relapse. Linear regression models were used to examine the effects of patient characteristics and immunophenotype on expression. The effects of expression and other patient characteristics on treatment outcomes were investigated using logistic (CR, RD) and proportional hazards (OS, RFS) regression analyses.

Multivariate analyses for the outcomes of OS, RFS, RD, and CR accounted for clinical and immunophenotype characteristics. Additional factors were excluded as needed to fit each model: expression of CD2, CD19, CD56, and HLA-DR on CR; expression of CD2, CD19, CD54, CD56, and HLA-DR on RD; expression of CD54 on OS; and expression of CD2, CD4, CD11a, CD16, CD19, C33, CD44, CD54, CD56, and HLA-DR on RFS.

Ingenuity pathway analysis (Qiagen Inc.) of target molecules identified for significant association with topo II α/β isoform expression was carried out using version 46901286 (11–21–2018). Ingenuity pathway analysis was carried out using: (a) drug pharmacodynamic targets, associated immunophenotype markers and topo II α/β; and (b) topo IIβ and significantly associated immunophenotype markers.
